# Risk Factors of Hypoperfusion on MRI of Ischemic Stroke Patients Within 7 Days of Onset

**DOI:** 10.3389/fneur.2021.668360

**Published:** 2021-05-07

**Authors:** Jingjing Xiao, Huazheng Liang, Yue Wang, Shaoshi Wang, Yi Wang, Yong Bi

**Affiliations:** ^1^Department of Neurology, Shanghai Fourth People's Hospital Affiliated to Tongji University School of Medicine, Shanghai, China; ^2^Department of Neurology, Translational Research Institute of Brain and Brain-Like Intelligence, Shanghai Fourth People's Hospital Affiliated to Tongji University School of Medicine, Shanghai, China; ^3^College of Pharmaceutical Sciences, Zhejiang University, Hangzhou, China

**Keywords:** ischemic stroke, magnetic resonance imaging, time to maximum of the residue function, risk factors, correlation analysis

## Abstract

**Objective:** Hypoperfusion is an important factor determining the prognosis of ischemic stroke patients. The present study aimed to investigate possible predictors of hypoperfusion on MRI of ischemic stroke patients within 7 days of stroke onset.

**Methods:** Ischemic stroke patients, admitted to the comprehensive Stroke Center of Shanghai Fourth People's Hospital affiliated to Tongji University within 7 days of onset between January 2016 and June 2017, were recruited to the present study. Magnetic resonance imaging (MRI), including both diffusion-weighted imaging (DWI) and perfusion-weighted imaging (PWI), was performed within 7 days of the symptom onset. Time to maximum of the residue function (*T*_*max*_) maps were automatically evaluated using the RAPID software. The volume of hypoperfusion was measured outside the infarct area based on ADC < 620 × 10^−6^ mm^2^/s. The 90 d mRS score was assessed through either clinic visits or telephone calls. Multivariate step-wise analysis was used to assess the correlation between MR findings and clinical variables, including the demographic information, cardio-metabolic characteristics, and functional outcomes.

**Results:** Among 635 patients admitted due to acute ischemic stroke within 7 days of onset, 241 met the inclusion criteria. Hypoperfusion volume of 38 ml was the best cut-off value for predicting poor prognosis of patients with cerebral infarction (90 d-mRS score ≥ 2). The incidences of MR perfusion *T*_*max*_ > 4–6 s maps with a volume of 0–38 mL or >38 mL were 51.9% (125/241) and 48.1% (116/241), respectively. Prior stroke and vascular stenosis (≥70%) were associated with MR hypoperfusion. Multivariate step-wise analysis showed that prior stroke and vascular stenosis (≥70%) were risk factors of *T*_*max*_ > 4–6 s maps, and the odds ratios (OR) were 3.418 (adjusted OR 95% CI: 1.537–7.600), and 2.265 (adjusted OR, 95% CI: 1.199–4.278), respectively.

**Conclusion:** Our results suggest that prior stroke and vascular stenosis (≥70%) are strong predictors of hypoperfusion in patients with acute ischemic stroke within 7 days of stroke onset.

## Introduction

Intravenous thrombolysis and endovascular therapy are effective methods for the treatment of acute ischemic stroke (1, 2). However, due to time window restrictions, only a small number of people receive timely treatment, and over 70% of stroke patients still have disabilities (modified Rankin classification, mRS2-6) due to the presence of hypoperfused tissues (3, 4). Quantitative assessment of hemodynamic indices of acute stroke patients will facilitate the discovery of potential predictors of hypoperfusion, which will reveal new targets for early and effective intervention. Currently, a number of factors have been reported to influence functional outcomes of acute ischemic stroke patients, including blood glucose, blood pressure, history of atrial fibrillation, baseline NIHSS, volume of core infarction, blood perfusion, and vascular lesions (5–12). Previous studies have shown that abnormal brain perfusion is closely related to stroke recurrence and functional outcome, but there are few studies on risk factors impacting brain perfusion.

*T*_*max*_ is a widely used parameter of magnetic resonance perfusion for patients with acute ischemic stroke and has been used in clinical trials (13, 14). Different *T*_*max*_ thresholds reflect different degrees of hypoperfused volumes, with a high threshold reflecting a low degree of hypoperfusion (15). Changes of *T*_*max*_ may reflect the microvascular integrity of collaterals and the perfusion status of brain tissue (16). In view of the fact that perfusion imaging is closely related to the status of collateral circulation, the cerebral perfusion parameters on MRI may be a good biomarker of collateral circulation. Therefore, it is reasonable to use *T*_*max*_ to evaluate the status of tissue hypoperfusion and facilitate decision-making on the choice of treatments for patients with AIS (17–19).

It is well-known that the penumbra is the area surrounding the ischemic core, which has a high risk of progressing to infarct. *T*_*max*_ > 6 s can accurately define the penumbra (20). Albers et al. screened patients with salvageable penumbra for endovascular thrombectomy using *T*_*max*_ > 6 s with the assistance of the RAPID software (17). Time to peak contrast concentration (TTP) or time at which the deconvolved residue function reaches its maximum value (*T*_*max*_) is generally used to evaluate hypoperfusion status. Compared with TTP, *T*_*max*_ has the advantage of reducing dependence on bolus shape and cardiac output (21). Therefore, *T*_*max*_ seems to be more appropriate in evaluating tissue lesions with hypoperfusion. It has been reported that *T*_*max*_ > 6 s or *T*_*max*_ > 4 s is more accurate than *T*_*max*_ > 2 s in predicting the salvageable penumbra or stroke progression. Difference between the volumes of *T*_*max*_ > 4 s and *T*_*max*_ > 6 s seems to be the best biomarker in identifying severe hypoperfusion (22). Studies have shown benefit from prolonged reperfusion therapy with increased likelihood of good prognosis through evaluating the ischemic penumbra with the perfusion parameter *T*_*max*_ (17, 19). However, few studies have reported risk factors of low perfusion in Chinese populations. Therefore, the present study aimed to quantitatively evaluate the hypoperfusion status of AIS patients and to explore the potential predictors of hypoperfusion on MRI.

## Materials and Methods

### Subjects

Acute ischemic stroke patients, admitted to the comprehensive Stroke Center of Shanghai Fourth People's Hospital affiliated to Tongji University within 7 days of onset between January 2016 and June 2017, were recruited to the present study.

The inclusion criteria were: (a) Patients who were admitted within 7 days of onset and evaluated by two stroke neurologists (23, 24); (b) MR images including both DWI and PWI were available at the time of hospitalization; (c) *T*_*max*_ maps were assessed using the RAPID software (iSchemaView USA, Version 4.9) (25) independently.

The exclusion criteria: patients did not have their perfusion status assessed using DWI and PWI within 7 days of stroke onset.

Demographic data, clinical variables, risk factors, neurologic deficits, time between MRI scan and stroke onset were documented for each patient. The 90 d mRS was evaluated by experienced neurologists.

### MRI Parameters

MRI scans were collected using a 1.5-T Avanto scanner (Siemens, Erlangen, Germany). The imaging protocol for this study included diffusion-weighted imaging (DWI), perfusion weighted imaging (PWI), apparent diffusion coefficient (ADC), fluid-attenuated inversion recovery (FLAIR), and magnetic resonance angiography (MRA). Imaging parameters were listed below, DWI: 19 slices, 192 × 192 matrices; slice thickness = 5.5 mm; TR/TE, 3,600/102 ms; field of view = 230 mm^2^, b = 0 and 1,000 s/mm^2^; EPI factor = 192; bandwidth = 964 Hz/pixel. FLAIR: 18 slices, 256 × 190 matrices; slice thickness = 5.5 mm; TR/TE, 4,000/92 ms; field of view = 230 mm^2^; TI = 1,532.6 ms; bandwidth = 190 Hz/Px; flip angle = 150°. Dynamic susceptibility contrast PWI (DSC-PWI): 19 slices, 128 × 128 matrices; slice thickness = 5 mm; TR/TE, 1,590/32 ms; measurements = 50; field of view = 230 mm^2^; band width = 1,346 Hz/pixel; flip angle = 90°. A Gd-DTPA contrast agent (gadopentetate dimeglumine injection; Shanghai Pharmaceutical Corporation, Shanghai, China) was injected intravenously (0.2 mmol/kg body weight) at a rate of 4 mL/s after a bolus with 30 ml normal saline. Three-dimensional time-of-flight MRA of the internal carotid artery (ICA) and intracranial circulation: 241 × 256 matrices, slice thickness = 0.7 mm; TR/TE, 25/7 ms; field of view = 180 mm^2^; Bandwidth = 100 Hz/PX; flip angle = 25°.

### Post-processing

Estimates of hypoperfusion on PWI were calculated using the RAPID software (iSchemaView USA, Version 4.9), which is an automated imaging post-processing system. ADC < 620 × 10^−6^ mm^2^/s was adopted to define the infarct core (26). Volumes of *T*_*max*_ > 4 s and >6 s were used to determine hypoperfusion in ischemic stroke patients. The volume of hypoperfusion was measured outside the infarct area, based on ADC < 620 × 10^−6^ mm^2^/s. Measurement of vascular stenosis was performed on Magnetic Resonance Angiography by two independent radiologists using the North American Symptomatic Carotid Endarterectomy Trial (NASCET) method (27). The extent of reduction of the internal arterial diameter was then graded. If the measurements of two radiologists were inconsistent, repetition will be required before reaching a conclusion.

### Clinical Outcomes

The primary outcome was assessed using the 90-day modified Rankin Scale (mRS). mRS ranges from 0 (asymptomatic) to 6 (death). Excellent functional outcomes are defined by a 90 d-mRS score ≤ 1, and poor functional outcomes are defined by a 90 d-mRS score ≥ 2. Three months after stroke onset, mRS scores were collected through clinic visits or telephone calls.

### Ethics

Ethical approval for this study was obtained from the Human Research Ethics Committee of Shanghai Fourth People's Hospital Affiliated to Tongji University School of Medicine. Written informed consent was obtained from all subjects.

### Statistical Analysis

Data analysis was performed using IBM SPSS (version 22.0) for Windows (SPSS Inc., Chicago, IL, USA). Continuous parameters were presented as mean ± standard deviation (SD) or median with interquartile range (IQR); categorical variables were summarized as independent proportions. Baseline information of patients with or without MRI perfusion abnormalities was compared using either *t*-test or Mann-Whitney U-test for continuous variables and χ^2^ or Fisher's exact test for categorical variables. In this study, the cutoff value was 38 ml for hypoperfusion. Logistic regression analysis was used to identify independent predictors of *T*_*max*_> 4–6 s maps. Multivariate step-wise regression modeling was used to correlate *T*_*max*_ > 4–6 s maps with potential risk factors with their *P*-values < 0.01. All correlation data were presented as odds ratios (OR) with their corresponding 95% confidence intervals (CI) and *P*-values. Statistical significance was considered when *P* < 0.05.

## Results

**Figure 1:**
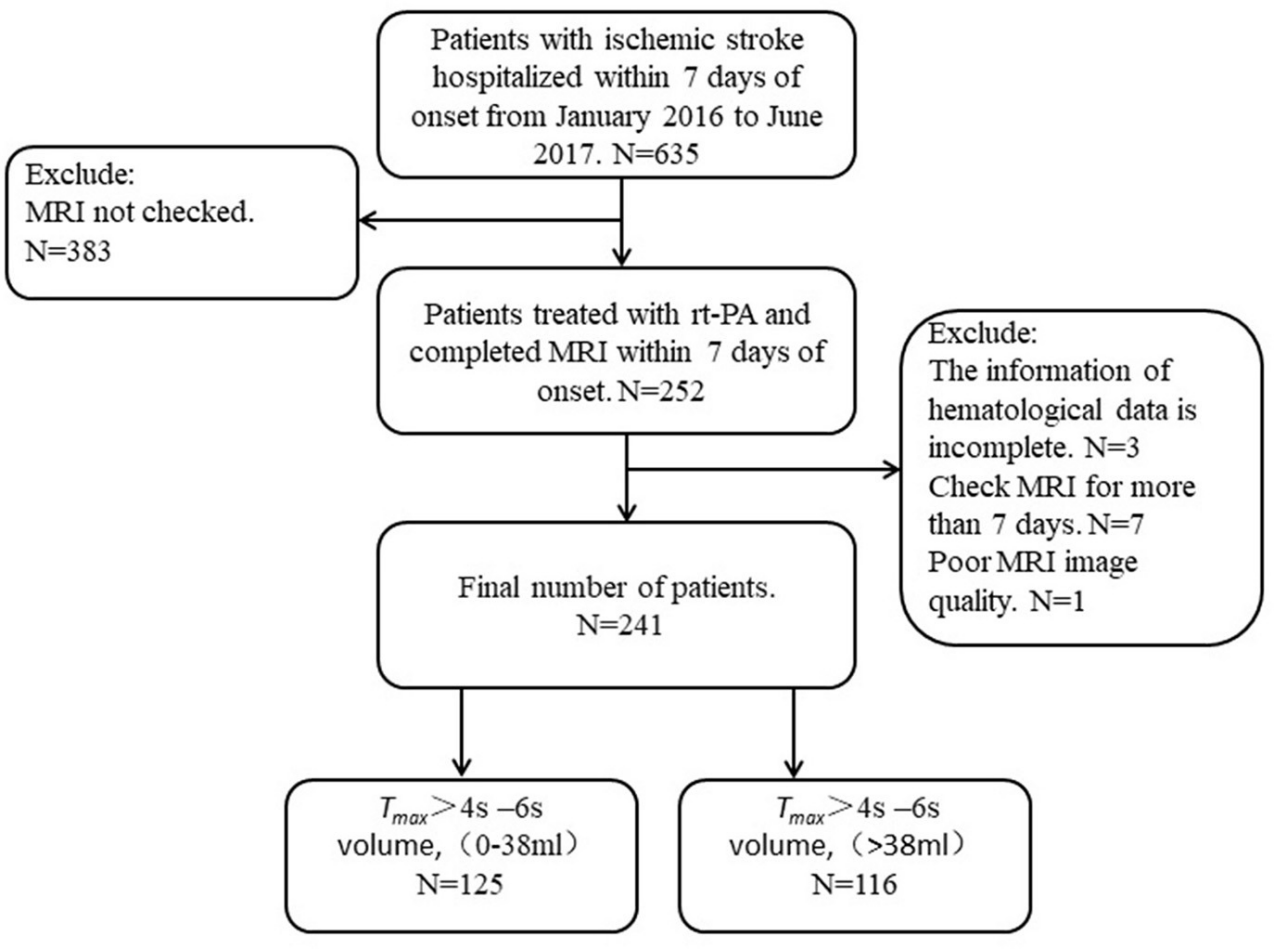
Flowchart of patient recruitment.

In the present study, 635 patients with acute ischemic stroke were admitted within 7 days of onset, but only 241 (73 women, 168 men; median age: 67 years) had technically adequate DWI and PWI scans. Among those 394 patients who were excluded, 383 of them did not have MRI, 3 could not provide the information of hematological data, 10 had their MRI more than 7 days after stroke onset, 1 had poor MRI image quality. Among these 241 patients, 107 patients had excellent functional outcomes and 134 poor functional outcomes. Among these patients, 125 had *T*_*max*_ > 4–6 s volume in the range of 0–38 ml and the other 116 had a volume > 38 ml (Figure 1, Table 1).

**Table 1:** Comparison of demographic and clinical characteristics among ischemic stroke patients within 7 days of onset based on *T*_*max*_ >4 s −6 s volume 38 ml.

**Characteristics**	**Total (*n* = 241)**	***T_***max***_* > 4–6 s volume, (0–38 ml) (*n* = 125)**	***T_***max***_* > 4–6 s volume, (>38 ml) (*n* = 116)**	***P*-value**
Age, y, median (IQR)	67 (61–79)	66 (59–75)	70 (62–80)	0.009
Male, *n* (%)	168 (69.7%)	86 (68.8%)	82 (70.7%)	0.75
**Medical history**, ***n*** **(%)**				
Hypertension	165 (68.5%)	84 (67.2%)	81 (69.8%)	0.661
Diabetes mellitus	83 (34.4%)	44 (35.2%)	39 (33.6%)	0.797
Atrial fibrillation	35 (14.5%)	9 (7.2%)	26 (22.4%)	0.001
Smoking	80 (33.2%)	39 (31.2%)	41 (35.3%)	0.495
Previous ischemic stroke	41 (17%)	12 (9.6%)	29 (25.0%)	0.001
**Cardo-metabolic**				
FBG, mmol/L, median (IQR)	6.0 (5.2–8.2)	5.9 (5.2–7.8)	6.1 (5.4–8.3)	0.310
TC, mmol/L, median (IQR)	3.72 (1.89–4.89)	3.73 (1.95–5.02)	3.68 (1.80–4.62)	0.385
LDL, mmol/L, median (IQR)	2.63(1.98–3.34)	2.65(2.0–3.40)	2.62(1.94–3.31)	0.466
TG, mmol/L, median (IQR)	2.32 (1.3–4.22)	2.35 (1.37–4.23)	2.24 (1.27–4.13)	0.408
HDL, mmol/L, median (IQR)	1.12 (0.88–1.34)	1.14 (0.91–1.33)	1.07 (0.85–1.39)	0.802
Hcy, umol/L, median (IQR)	13.6 (11.3–16.6)	12.4(10.9–15.85)	14.25 (12.0–17.0)	0.011
SBP at baseline, mm Hg, median (IQR)	140 (130–160)	150 (135–160)	140 (130–153)	0.093
DBP at baseline, mm Hg, median (IQR)	80 (78–90)	80 (79–90)	80 (77–89)	0.404
Admission NIHSS score, median (IQR)	3 (1–9)	3 (1–6)	6 (2–10)	<0.001
Treatment				0.782
Alteplase treatment	36 (14.9%)	19 (15.2%)	17 (14.7%)	
Bridge-EVT	2 (0.8%)	2 (1.6%)	0	
Direct-EVT	12 (5.0%)	1 (0.8%)	11 (9.5%)	
Standard medical therapy	191 (79.3%)	103 (82.4%)	88 (75.9%)	
Symptom onset to the MR perfusion, d, median (IQR)	3 (1–6)	4 (2–6)	3 (1–6)	0.006
Vascular stenosis (≥70%), *n* (%)	154 (63.9%)	62 (49.6%)	92 (79.3%)	<0.001
CD4/CD8 (≥1.7), *n* (%)	99 (41.1%)	58 (46.4%)	41 (35.3%)	0.081
Secondary bleeding, *n* (%)	12 (9%)	6 (11.5%)	6 (7.4%)	0.616
	7 (2.9%)	2 (1.6%)	5 (4.3%)	0.266
Clinical outcomes (90 d mRS)	2 (0–3)	1 (0–3)	2 (1–4)	<0.001
Excellent functional outcome (0–1)	107 (44.4%)	73 (58.4%)	34 (29.3%)	
Poor functional outcome (2–6)	134 (55.6%)	52 (41.6%)	82 (70.7%)	
ADC <620 × 10^−6^ mm^2^/s volume, ml, median (IQR)	0 (0–7.5)	0 (0–0)	0 (0–24.5)	<0.001

*Measurement data conformed to normal distribution variables expressed as mean ± standard deviation; non-normal distribution variables were expressed as median (25–75%). The categorical variables are expressed in terms of frequency (percentage)*.

*IQR, interquartile range; INR, International standardization ratio; LDL, Low-density lipoprotein; HDL, Low-density lipoprotein; NIHSS, National Institutes of Health Stroke Scale; SBP, systolic blood pressure; DBP, diastolic blood pressure; TC, total cholestrol; Hcy, homocysteine; FBG, fasting blood-glucose; IVT, intravenous thrombolysis; Direct-EVT, direct endovascular thrombectomy; Bridge-EVT, bridge endovascular thrombectomy; mRS, modified Rankin Scale; T_max_ > 4 s, Time to maximum of the residue function > 4 s; T_max_ > 6 s, Time to maximum of the residue function > 6 s; ADC, apparent diffusion coefficient; T_max_ > 4–6 s volume, Volume difference between T_max_ > 4 s and T_max_ > 6 s*.

*Significant difference at α < 0.05*.

### Baseline Characteristics

Baseline characteristics of patients included in the present study were shown in Tables 1, 2. The median (IQR) age of these patients was 67 (61–79) years and their median Admission NIHSS score was 3 (IQR: 1–9). Perfusion status was evaluated after a median (IQR) delay of 3 (1–6) days from the symptom onset. The median ADC volume was 0 (IQR: 0–7.5) ml. The median fasting blood glucose (FBG) was 6.0 (IQR: 5.2–8.2) mmol/L. A history of hypertension was present in 68.5% (165/241) of patients, diabetes mellitus in 34.4% (83/241), atrial fibrillation in 14.5% (35/241), prior stroke in17% (41/241) and smoking in 33.2% (80/241). The patients were divided into four groups: intravenous thrombolysis (36/241), Bridge endovascular thrombectomy (2/241), Direct endovascular thrombectomy (12/241), and standard medical therapy alone (191/241) (Table 1).

**Table 2:** Imaging characteristics of ischemic stroke patients with excellent and poor functional outcomes.

**Characteristics**	**Total (*n* = 241)**	**Excellent functional outcome group (90 d mRS 0–1) (*n* = 107)**	**Poor functional outcome group (90 d mRS 2–6) (*n* = 134)**	***P*-value**
Vascular stenosis (≥70%), *n* (%)	154 (63.9%)	63 (58.9%)	91 (67.9%)	0.147
ADC <620 × 10^−6^ mm^2^/s volume, ml, median (IQR)	0 (0–8)	0 (0–0)	0 (0–12.3)	<0.001
*T_*max*_* > 4 s volume, ml, median(IQR)	35 (8–105)	19 (5–63)	71 (14.0–166.3)	<0.001
*T_*max*_* > 6 s volume, ml, median(IQR)	0 (0–13)	0 (0–3)	0 (0–32.5)	<0.001
*T_*max*_* > 4–6 s volume, ml, median(IQR)	35 (8–88)	18 (4–57)	48 (14–113)	<0.001
*T_*max*_* > 4 s –ADC volume, ml, median (IQR)	34 (6–95)	18 (0–63)	54 (10.8–146.0)	<0.001
*T_*max*_* > 6 s –ADC volume, ml, median (IQR)	0 (0–3.5)	0 (0–0)	0 (0–13.5)	0.004

*Measurement data conformed to normal distribution variables expressed as mean ± standard deviation; non-normal distribution variables were expressed as median (25–75%). The categorical variables are expressed in terms of frequency (percentage)*.

*IQR, interquartile range; mRS, modified Rankin Scale; ADC, apparent diffusion coefficient; T_max_ > 4 s, Time to maximum of the residue function > 4 s; T_max_ > 6 s, Time to maximum of the residue function > 6 s; T_max_ > 4–6 s volume, Volume difference between T_max_ > 4 s and T_max_ > 6 s; T_max_ > 4 s–ADC volume, Volume difference between T_max_ > 4 s and ADC; T_max_ > 6 s–ADC volume, Volume difference between T_max_ > 6 s and ADC*.

*Significant difference at α < 0.05*.

### Comparison of Demographic and Clinical Characteristics Among Ischemic Stroke Patients Within 7 Days of Onset Based on *T_*max*_* > 4–6 s Volume 38 ml

**Figure 2:**
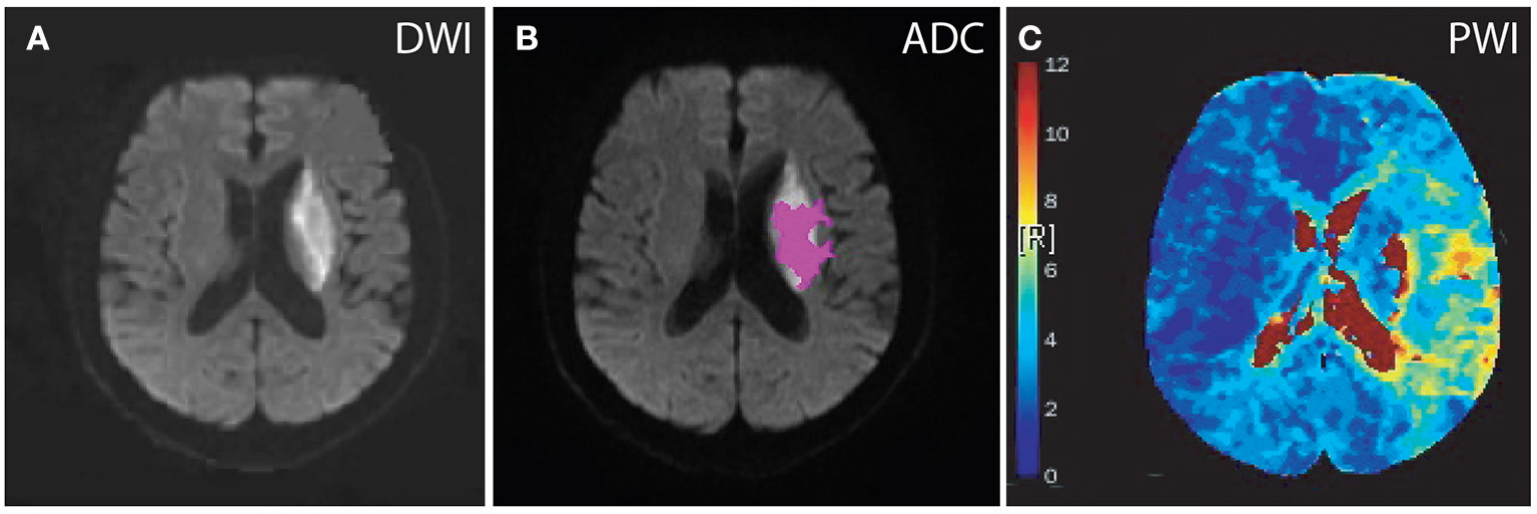
Diffusion and perfusion abnormalities of a patient treated with intravenous tPA. A 63-year-old male who presented with dysarthria and weakness in the right arm, hypertension for more than 3 years, vascular stenosis (≥70%), no previous ischemic stroke, MRI scan was completed 6 days after stroke onset. DWI, diffusion-weighted imaging; PWI, perfusion-weighted imaging; ADC, apparent diffusion coefficient; *T*_*max*_, Time to maximum of the residue function. *T*_*max*_ color scale: 4 s < *T*_*max*_ ≤ 6 s (blue); 6 s < *T*_*max*_ ≤ 8 s (green); 8 s < *T*_*max*_ ≤ 10 s (yellow); 10 s < *T*_*max*_ (red). **(A)** DWI, lesion volume was 22 ml. **(B)** ADC, lesion volume was 9 ml. **(C)** PWI, lesion volumes according to *T*_*max*_ delay were as follows: *T*_*max*_ > 4 s, 132 ml; *T*_*max*_ > 4 s–ADC volume, 123 ml; *T*_*max*_ > 6 s, 22 ml; *T*_*max*_ > 6 s–ADC volume, 13 ml; *T*_*max*_ > 8 s, 4 ml; and *T*_*max*_ > 10 s, 3 ml.

**Figure 3:**
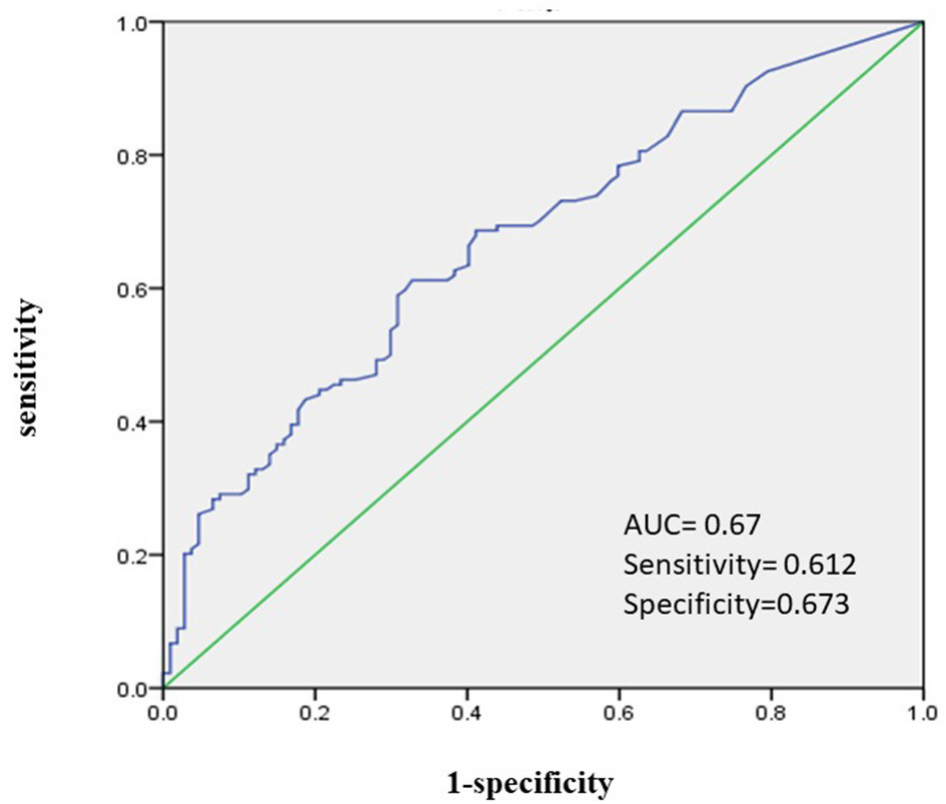
Area under the ROC curve predicts hypoperfusion based on 90 d-mRS.

Demographic characteristics, possible risk factors associated with the *T*_*max*_ > 4–6 s map, and comparisons of these variables between groups with volume = 0–38 ml and >38 ml were presented in Table 1. Hypoperfusion volume of 38 ml was the best cut-off value for predicting poor prognosis of patients with cerebral infarction (90 d-mRS score ≥ 2) as shown by the ROC (AUC: 0.67, 95% CI: 0.603–0.738, sensitivity: 0.612, specificity 0.673, *p* < 0.001) (Figures 2, 3). In univariate analyses, factors associated with hypoperfusion were: age [66 (59–75) vs. 70 (62–80), *p* = 0.009], atrial fibrillation (7.2 vs. 22.4%, *p* = 0.001), previous ischemic stroke (9.6 vs. 25.0%, *p* = 0.001), homocysteine [12.4 (10.9–15.85) vs. 14.25 (12.0–17.0), *p* = 0.011], admission NIHSS score [3 (1–6) vs. 6 (2–10), *p* < 0.001], symptom onset to the MR perfusion [4 (2–6) vs. 3 (1–6), *p* = 0.006], vascular stenosis (≥70%) (49.6 vs. 79.3%, *p* < 0.001), ADC volume [0 (0–0) vs. 0 (0–24.5), *p* < 0.001]. Other variables were not statistically different (Table 1).

Excellent functional outcomes (mRS 0–1) were present in 44.4% (107/241) of patients, among whom the median (IQR) *T*_*max*_ > 4–6 s volume, *T*_*max*_ > 6 s volume, and *T*_*max*_ > 4 s volume were 18 (4–57) ml, 0 (0–3) ml, and 19 (5–63) ml, respectively. MRA scans with adequate quality of both the carotid and intracranial vessels were available in the present study. MRA showed that vascular stenosis (>70%) was detected in 154 (63.9%) patients (Table 2).

### Prediction of MRI Perfusion Abnormality

In the present study, we divided patients with hypoperfusion into two groups based on the volume of *T*_*max*_ > 4–6 s: *T*_*max*_ > 4–6 s = 0–38 ml and *T*_*max*_ > 4–6 s > 38 ml. The incidences of MR perfusion *T*_*max*_ > 4–6 s = 0–38 ml and *T*_*max*_ > 4–6 s > 38 ml were 51.9% (125/241) and 48.1% (116/241), respectively (Table 1).

In the univariate binary logistic regression analysis, age (*P* = 0.010, OR = 1.031, 95% CI: 1.007–1.056), atrial fibrillation (*P* = 0.001, OR = 3.723, 95% CI: 1.662–8.340), previous ischemic stroke (*P* = 0.002, OR = 3.139, 95% CI: 1.515–6.504), admission NIHSS score (*P* < 0.001, OR = 1.091, 95% CI: 1.040–1.145), symptom onset to MR perfusion (*P* = 0.018, OR = 0.872, 95% CI: 0.779–0.977), vascular stenosis (≥70%) (*P* < 0.001, OR = 3.895, 95% CI: 2.203–6.887), ADC volume (*P* < 0.001, OR = 1.044, 95% CI: 1.019–1.070) were independently associated with MR perfusion abnormality in ischemic stroke patients within 7 days of onset (Table 3).

**Table 3:** Factors independently associated with *T*_*max*_ > 4–6 s > 38 mL in ischemic stroke patients within 7 days of onset.

	**OR (95% CI)**	***P*-value**	**Adjusted OR (95% CI)**	***P*-value**
Age	1.031 (1.007–1.056)	0.010	1.037 (1.009–1.066)	0.010
Atrial fibrillation	3.723 (1.662–8.340)	0.001	1.404 (0.545–3.614)	0.482
Previous ischemic stroke	3.139 (1.515–6.504)	0.002	3.418 (1.537–7.600)	0.001
Admission NIHSS score	1.091 (1.040–1.145)	<0.001	1.038 (0.983–1.096)	0.181
symptom onset to the MR perfusion	0.872 (0.779–0.977)	0.018	0.905 (0.793–1.034)	0.142
Vascular stenosis (≥70%)	3.895 (2.203–6.887)	<0.001	2.265 (1.199–4.278)	0.012
ADC <620 × 10^−6^ mm^2^/s volume	1.044 (1.019–1.070)	<0.001	1.033 (1.009–1.058)	0.008
HCY	1.007 (0.986–1.029)	0.516	N	N

*CI, confidence interval; OR, odds ratio; NIHSS, National Institutes of Health Stroke Scale; SBP, systolic blood pressure; ADC, apparent diffusion coefficient, Hcy, homocysteine*.

Multivariate step-wise regression modeling was performed for predictors with *P*-values < 0.01 and the multivariate logistic regression analysis performed to show the correlation between previous ischemic stroke, stenosis (≥70%), and MR perfusion abnormalities. Patients with previous ischemic stroke and stenosis were likely to develop hypoperfusion on PWI maps. The adjusted odds ratios were 3.418 (95% CI: 1.537–7.600, *P* = 0.001) and 2.265 (95% CI: 1.199–4.278, *P* = 0.012), respectively. Other variables were not significantly associated with hypoperfusion (Table 3).

## Discussion

In the present study, risk factors of hypoperfusion on MRI were analyzed for AIS patients admitted within 7 days of onset. It was found that the perfusion parameter *T*_*max*_ > 4–6 s volume was related to clinical prognosis. Patients with previous stroke and vascular stenosis (≥70%) were more likely to have hypoperfusion, and these two were independent risk factors of low perfusion as shown by *T*_*max*_ > 4–6 s map > 38 ml.

### *T_*max*_* > 4–6 s Map and 90 d mRS

It is well-known that DWI and ADC maps were closely related to the final infarct volume and were important predictors of clinical prognosis (9, 28). The present study used non-invasive multimode magnetic resonance imaging to quantitatively evaluate *T*_*max*_ > 4–6 s map. It was found that the tissue perfusion status of *T*_*max*_ > 4–6 s was closely related to 90 d mRS. The greater the *T*_*max*_ > 4–6 s map was, the worse the 90 d mRS was. Previous studies have shown that perfusion imaging was closely related to the status of collateral circulation, and the cerebral perfusion parameter *T*_*max*_ was a good biomarker of collateral volume (29). Therefore, it is reasonable to use the *T*_*max*_ > 4–6 s map to evaluate tissue hypoperfusion.

Collateral status could be used to predict the prognosis of patients with acute ischemic stroke as the key determinant (30, 31). Perfusion status changes temporally and spatially. Findings from perfusion imaging reflect collateral status or response to treatment for those without large vessel occlusion within 6 h or for those with large vessel occlusion within 24 h. These are closely correlated with clinical prognosis. In this study, the cutoff value 38 ml of *T*_*max*_ > 4–6 s map was used to define hypoperfusion on MRI with an aim to find risk factors that were related to hypoperfusion, and to provide a reasonable direction for accurate control of these risk factors. This would improve clinical prognosis and reduce the occurrence of recurrent ischemic events.

### Previous Ischemic Stroke and *T_*max*_* > 4–6 s Maps

This study found that hypoperfusion was associated with recurrent stroke and persistent deterioration of neurological functions. It might be related to a certain proportion of vascular stenosis and distal hypoperfusion in patients with previous strokes, which are likely to recur. This is consistent with the result of a previous studies (32). Previous studies have shown that stroke patients have a recurrence rate of 17% within 1 year. In symptomatic intracranial atherosclerotic stenosis (ICAS) patients, the more severe the baseline hypoperfusion was, the higher the risk of stroke recurrence was (33–35). In this study, 41 patients had a history of stroke, among whom 29 had their *T*_*max*_ > 4–6 s volume > 38 ml, accounting for 25% of this type of patients. Among these 41 patients, 30 had vascular stenosis ≥ 70%, accounting for 73.2% of these patients. Previous strokes are a risk factor of hypoperfusion. The possible reason is that the majority of patients with previous strokes have vascular stenosis, which leads to distal hypoperfusion, and consequently recurrence of ischemic stroke.

### Vascular Stenosis and *T_*max*_* > 4–6 s Map

Spencer and Reid first proposed the relationship between cerebral artery stenosis and cerebral blood flow, which predicted a decrease in blood flow when stenosis was >70% (36, 37). Cerebral artery stenosis or occlusion can trigger serious hemodynamic disorders. However, it has been shown that the severity of vascular stenosis does not necessarily affect the status of distal blood flow (38). In this study, it was found that vascular stenosis was related to the *T*_*max*_ > 4–6 s map. 63.9% of the patients had evidence of ipsilateral proximal artery stenosis or occlusion on MRA, among whom the proportion of tissue hypoperfusion was 60.4% (93/153). Therefore, tissue hypoperfusion may be more likely to occur in patients with ipsilateral proximal artery stenosis or occlusion on MRA, which is consistent with previous studies (39, 40). This might be due to changes in the cerebral vascular structure and function resulted from intracranial atherosclerosis. Vascular stenosis affects the hemodynamic status, mainly through the decrease of cerebrovascular reserve. In the presence of insufficient collaterals, the decrease in pressure may lead to hypoperfusion.

The volume of hypoperfusion at different thresholds of *T*_*max*_ non-invasively reflected the status of collaterals, which is consistent with findings of previous studies. Therefore, it is likely that perfusion parameters on MRI will be a good biomarker for volumes of collateral blood flow (29). The benefit of the present study is to non-invasively assess hypoperfusion volumes at the early stage of ischemic stroke, which reflects the collateral status. It will provide evidence for early intervention to halt stroke progression, prevent stroke recurrence, and to improve clinical prognosis.

The present study has a number of limitations. Firstly, it is a retrospective study in which all subjects were recruited from a local hospital, which may result in selection bias. In addition, it has a relatively small sample size and the conclusion from this study may not be extrapolated to all ischemic stroke patients. Therefore, prospective studies with a large sample size are required to confirm our findings. Secondly, it is a cross-sectional study and can not pinpoint the direct causality between hypoperfusion and the risk factors of ischemic stroke patients within 7 days of onset. A longitudinal design can help to investigate the direct causal relationship between risk factors and MR hypoperfusion in future studies. Thirdly, *T*_*max*_ > 4–6 s and volume of hypoperfusion > 38 ml were used to determine hypoperfusion,which is based on the cutoff value of 38 ml in our analysis, whether this method has better accuracy and applicability needs to be verified by future prospective, large-scaled studies. Fourthly, The present study did not repeat MR scan between the therapeutic window and 90 days to evaluate the perfusion status, which might result in treatment bias.

In conclusion, hypoperfusion could be found in ischemic stroke patients within 7 days of onset when PWI was examined, which is related to clinical prognosis. Patients with previous ischemic strokes and vascular stenosis are more likely to have severe hypoperfusion and poor functional outcomes. Accurate control of risk factors may effectively improve functional outcomes. However, larger prospective studies are needed to confirm these findings.

## Data Availability Statement

The original contributions presented in the study are included in the article/supplementary material, further inquiries can be directed to the corresponding author/s.

## Ethics Statement

The studies involving human participants were reviewed and approved by Human Research Ethics Committee of Shanghai Fourth People's Hospital Affiliated to Tongji University School of Medicine. The patients/participants provided their written informed consent to participate in this study.

## Author Contributions

YB conceived and designed this study. JX and HL collected data and performed statistical analysis and drafted the manuscript. YueW helped with data collection and data analysis. YiW and YB revised the manuscript. All authors contributed to the article and approved the submitted version.

## Conflict of Interest

The authors declare that the research was conducted in the absence of any commercial or financial relationships that could be construed as a potential conflict of interest.

## References

[1] The National Institute of Neurological Disorders, and Stroke rt-PA Stroke Study Group. Tissue plasminogen activator for acute ischemic stroke. N Engl J Med. (1995) 333:1581–7. 10.1056/NEJM199512143332401 7477192

[2] Goyal M Demchuk AM Menon BK Eesa M Rempel JL Thornton J. Randomized assessment of rapid endovascular treatment of ischemic stroke. N Engl J Med. (2015) 372:1019–30. 10.1056/NEJMoa1414905 25671798

[3] Bhatia R Hill MD Shobha N Menon B Bal S Kochar P. Low rates of acute recanalization with intravenous recombinant tissue plasminogen activator in ischemic stroke: real-world experience and a call for action. Stroke. (2010) 41:2254–8. 10.1161/STROKEAHA.110.592535 20829513

[4] Saqqur M Uchino K Demchuk AM Molina CA Garami Z Calleja S. Site of arterial occlusion identified by transcranial Doppler predicts the response to intravenous thrombolysis for stroke. Stroke. (2007) 38:948–54. 10.1161/01.STR.0000257304.21967.ba 17290031

[5] Huang J Zhang X Li J Tang L Jiao X Lv X. Impact of glucose fluctuation on acute cerebral infarction in type 2 diabetes. Can J Neurol Sci. (2014) 41:486–92. 10.1017/S0317167100018539 24878474

[6] Stead LG Gilmore RM Vedula KC Weaver AL Decker WW Brown RD Jr. Impact of acute blood pressure variability on ischemic stroke outcome. Neurology. (2006) 66:1878–81. 10.1212/01.wnl.0000219628.78513.b5 16801654

[7] Tu HT Campbell BC Christensen S Desmond PM De Silva DA Parsons MW. Worse stroke outcome in atrial fibrillation is explained by more severe hypoperfusion, infarct growth and hemorrhagic transformation. Int J Stroke. (2015) 10:534–40. 10.1111/ijs.12007 23489996 PMC3688700

[8] Ali SF Siddiqui K Ay H Singhal A Viswanathan A Rost N. Baseline predictors of poor outcome in patients too good to treat with intravenous thrombolysis. Stroke. (2016) 47:2986–92. 10.1161/STROKEAHA.116.014871 27834750

[9] Wheeler HM Mlynash M Inoue M Tipirneni A Liggins J Zaharchuk G. Early diffusion-weighted imaging and perfusion-weighted imaging lesion volumes forecast final infarct size in DEFUSE 2. Stroke. (2013) 44:681–5. 10.1161/STROKEAHA.111.000135 23390119 PMC3625664

[10] Tomsick T. TIMI, TIBI, TICI: I came, I saw, I got confused. AJNR Am J Neuroradiol. (2007) 28:382–4. 17297017 PMC7977395

[11] Pan JW Yu XR Zhou SY Wang JH Zhang J Geng DY. Computed tomography perfusion and computed tomography angiography for prediction of clinical outcomes in ischemic stroke patients after thrombolysis. Neural Regen Res. (2017) 12:103–8. 10.4103/1673-5374.198994 28250755 PMC5319214

[12] Soares BP Tong E Hom J Cheng SC Bredno J Boussel L. Reperfusion is a more accurate predictor of follow-up infarct volume than recanalization: a proof of concept using CT in acute ischemic stroke patients. Stroke. (2010) 41:e34–40. 10.1161/STROKEAHA.109.568766 19910542 PMC2909663

[13] Inoue M Mlynash M Straka M Kemp S Jovin TG Tipirneni A. Clinical outcomes strongly associated with the degree of reperfusion achieved in target mismatch patients: pooled data from the diffusion and perfusion imaging evaluation for understanding stroke evolution studies. Stroke. (2013) 44:1885–90. 10.1161/STROKEAHA.111.000371 23704106 PMC3810454

[14] De Silva DA Brekenfeld C Ebinger M Christensen S Barber PA Butcher KS. The benefits of intravenous thrombolysis relate to the site of baseline arterial occlusion in the echoplanar imaging thrombolytic evaluation trial (EPITHET). Stroke. (2010) 41:295–9. 10.1161/STROKEAHA.109.562827 20056931

[15] Lansberg MG Lee J Christensen S Straka M De Silva DA Mlynash M. RAPID automated patient selectionfor reperfusion therapy a pooled analysis of the echoplanar imaging thrombolytic evaluation trial (EPITHET) and the diffusion and perfusion imaging evaluation for understanding stroke evolution (DEFUSE) study. Stroke. (2011) 42:1608–14. 10.1161/STROKEAHA.110.609008 21493916 PMC3104106

[16] Calamante F Christensen S Desmond PM Ostergaard L Davis SM Connelly A. The physiological significance of the time-to-maximum (Tmax) parameter in perfusion MRI. Stroke. (2010) 41:1169–74. 10.1161/STROKEAHA.110.580670 20413735

[17] Albers GW Marks MP Kemp S Christensen S Tsai JP Ortega-Gutierrez S. Thrombectomy for stroke at 6 to 16 hours with selection by perfusion imaging. N Engl J Med. (2018) 378:708–18. 10.1056/NEJMoa1713973 29364767 PMC6590673

[18] Nogueira RG Jadhav AP Haussen DC Bonafe A Budzik RF Bhuva P. Thrombectomy 6 to 24 hours after stroke with a mismatch between deficit and infarct. N Engl J Med. (2018) 378:11–21. 10.1056/NEJMoa1706442 29129157

[19] Ma H Campbell BCV Parsons MW Churilov L Levi CR Hsu C. Thrombolysis guided by perfusion imaging up to 9 hours after onset of stroke. N Engl J Med. (2019) 380:1795–803. 10.1056/NEJMoa1813046 31067369

[20] Olivot JM Mlynash M Thijs VN Purushotham A Kemp S Lansberg MG. Relationships between cerebral perfusion and reversibility of acute diffusion lesions in DEFUSE: insights from RADAR. Stroke. (2009) 40:1692–7. 10.1161/STROKEAHA.108.538082 19299632 PMC2709804

[21] Lansberg MG Straka M Kemp S Mlynash M Wechsler LR Jovin TG. Magnetic resonance imaging profile and response to endovascular reperfusion: results of the DEFUSE 2 prospective cohort study. Lancet Neurol. (2012) 11:860–7. 10.1016/S1474-4422(12)70203-X 22954705 PMC4074206

[22] Olivot JM Mlynash M Thijs VN Kemp S Lansberg MG Wechsler L. Optimal Tmax threshold for predicting penumbral tissue in acute stroke. Stroke. (2009) 40:469–75. 10.1161/STROKEAHA.108.526954 19109547 PMC2670783

[23] Powers WJ Zivin J. Magnetic resonance imaging in acute stroke not ready for prime time. Neurology. (1998) 50:842–3. 10.1212/WNL.50.4.842 9566357

[24] Tourdias T Renou P Sibon I Asselineau J Bracoud L Dumoulin M. Final cerebral infarct volume is predictable by MR imaging at 1 week. AJNR Am J Neuroradiol. (2011) 32:352–8. 10.3174/ajnr.A2271 20966063 PMC7965712

[25] Straka M Albers GW Bammer R. Real-time diffusionperfusion mismatch analysis in acute stroke. J Magn Reson Imaging. (2010) 32:1024–37. 10.1002/jmri.22338 21031505 PMC2975404

[26] Purushotham A Campbell BC Straka M Mlynash M Olivot JM Bammer R. Apparent diffusion coefficient threshold for delineation of ischemic core. Int J Stroke. (2015) 10:348–53. 10.1111/ijs.12068 23802548 PMC3786020

[27] North American Symptomatic Carotid Endarterectomy Trial Collaborators Barnett HJM Taylor DW Haynes RB Sackett DL Peerless SJ. Beneficial effect of carotid endarterectomy in symptomatic patients with high-grade carotid stenosis. N Engl J Med. (1991) 325:445–3. 10.1056/NEJM199108153250701 1852179

[28] Warach S Gaa J Siewert B Wielopolski P Edelman RR. Acute human stroke studied by whole brain echo planar diffusion-weighted magnetic resonance imaging. Ann Neurol. (1995) 37:231–41. 10.1002/ana.410370214 7847864

[29] Cortijo E Calleja AI Garciá-Bermejo P Mulero P Pérez-Fernández S Reyes J. Relative cerebral blood volume as a marker of durable tissue-at-risk viability in hyperacute ischemic stroke. Stroke. (2014) 45:113–8. 10.1161/STROKEAHA.113.003340 24281229

[30] Vagal A Aviv R Sucharew H Reddy M Hou Q Michel P. Collateral clock is more important than time clock for tissue fate. Stroke. (2018) 49:2102–7. 10.1161/STROKEAHA.118.021484 30354992 PMC6206882

[31] Arenillas JF Cortijo E García-Bermejo P Levy EI Jahan R Liebeskind D. Relative cerebral blood volume is associated with collateral status and infarct growth in stroke patients in SWIFT PRIME. J Cereb Blood Flow Metab. (2018) 38:1839–47. 10.1177/0271678X17740293 29135347 PMC6168913

[32] De Havenon A Khatri P Prabhakaran S Yeatts SD Peterson C Sacchetti D. Hypoperfusion distal to anterior circulation intracranial atherosclerosis is associated with recurrent stroke. J Neuroimaging. (2020) 30:468–70. 10.1111/jon.12710 32579278 PMC8010594

[33] Wang Y Zhao X Liu L Soo YO Pu Y Pan Y. Prevalence and outcomes of symptomatic intracranial large artery stenoses and occlusions in China: the Chinese Intracranial Atherosclerosis (CICAS) Study. Stroke. (2014) 45:663–9. 10.1161/STROKEAHA.113.003508 24481975

[34] Kasner SE Chimowitz MI Lynn MJ Howlett-Smith H Stern BJ Hertzberg VS. Predictors of ischemic stroke in the territory of a symptomatic intracranial arterialstenosis. Circulation. (2006) 113:555–63. 10.1161/CIRCULATIONAHA.105.578229 16432056

[35] Lyu J Ma N Tian C Xu F Shao H Zhou X. Perfusion and plaque evaluation to predict recurrent stroke in symptomatic middle cerebral artery stenosis. Stroke Vasc Neurol. (2019) 4:129–34. 10.1136/svn-2018-000228 31709118 PMC6812634

[36] Spencer MP Reid JM. Quantitation of carotid stenosis with continuouswave (C-W) Doppler ultrasound. Stroke. (1979) 10:326–30. 10.1161/01.STR.10.3.326 462521

[37] Carrera E Lee LK Giannopoulos S Marshall RS. Cerebrovascular reactivityand cerebral autoregulation in normal subjects. J Neurol Sci. (2009) 285:191–4. 10.1016/j.jns.2009.06.041 19608202

[38] Powers WJ Press GA Grubb RL Jr Gado M Raichle ME. The effect of hemodynamically significant carotid artery disease on the hemodynamic status of the cerebral circulation. Ann Intern Med. (1987) 106:27–35. 10.7326/0003-4819-106-1-27 3491558

[39] Ge X Zhao H Zhou Z Li X Sun B Wu H. Association of fractional flow on 3D-TOF-MRA with cerebral perfusion in patients with MCA stenosis. AJNR Am J Neuroradiol. (2019) 40:1124–31. 10.3174/ajnr.A6095 31196857 PMC7048535

[40] Lu SS Ge S Su CQ Xie J Mao J Shi HB. MRI of plaque characteristics and relationship with downstream perfusion and cerebral infarction in patients with symptomatic middle cerebral artery stenosis. J Magn Reson Imaging. (2018) 48:66–73. 10.1002/jmri.25879 29083523

